# Local self governance in health - a study of it’s functioning in Odisha, India

**DOI:** 10.1186/s12913-016-1785-8

**Published:** 2016-10-31

**Authors:** Bhuputra Panda, Sanjay P. Zodpey, Harshad P. Thakur

**Affiliations:** 1 Public Health Foundation of India, IIPH-Bhubaneswar, Bhubaneswar, India; 2 Public Health Foundation of India, New Delhi, India; 3 School of Health Systems Studies, TISS, Mumbai, India

**Keywords:** Local decision making, Rogi kalyan samiti, Odisha, Health system functioning, Perception, Experience

## Abstract

**Background:**

Local decision making is linked to several service quality improvement parameters. Rogi Kalyan Samitis (RKS) at peripheral decision making health units (DMHU) are composite bodies that are mandated to ensure accountability and transparency in governance, improve quality of services, and facilitate local responsiveness. There is scant literature on the nature of functioning of these institutions in Odisha. This study aimed to assess the perception of RKS members about their roles, involvement and practices with respect to local decision making and management of DMHUs; it further examined perceptual and functional differences between priority and non-priority district set-ups; and identified predictors of involvement of RKS members in local governance of health units.

**Methods:**

As members of RKS, health service providers, officials in administrative/managerial role, elected representatives, and officials from other departments (including independent members) constituted our study sample. A total of 112 respondents were interviewed across 6 districts, through a multi-stage stratified random sampling; we used a semi-structured interview schedule that comprised mainly of close-ended and some open-ended questions. Descriptive and inferential statistics were used to compare 3 priority (PD) and 3 non-priority districts (NPD), categorized on the basis of Infant Mortality Rate (IMR) estimates of 2011 as proxy of population health. Governance, human resource management, financial management and quality improvement functions were studied in detail. Opinion about various individual and organizational factors in local self-governance and predictors of involvement were identified.

**Results:**

The socio-demographic profile and composition of respondents were comparable between PD and NPD. Majority of respondents were ‘satisfied’ with their current roles in the governance of local health institutions. About one-fourth opined that the amount of funds allocated to RKS under National Health Mission (NHM) was ‘grossly insufficient’. Fifty percent of respondents said they requested for additional funds, last year, and 38.8 % informed that they requested additional funds for purchase of drugs. About 87 % respondents were satisfied with their role in the local governance of the health units (PD = 94.3 % vs. NPD = 80.7 %). Almost all (PD = 98 % vs. NPD = 80.7 %) opined that local decision making helped in improving the performance of health units. For most of the open-ended questions the responses were non-specific. Staggering differences were found between PD and NPD with respect to their involvement in district plan preparation (NPD = 78.9 % vs. PD = 58.5 %), training in plan preparation (NPD = 47.4 % vs. PD = 27.5 %), participation of officials from other departments (PD = 96.9 % vs. NPD = 45.5 %), and inclusion of activities of other sectors (PD = 70.8 % vs. NPD = 41.8 %). Whereas, no significant PD-NPD difference was found about their perceived ‘involvement’ in undertaking the 12 designated responsibilities. Composite scores on various individual and organizational factors were compared and found to be varying significantly. Through regression, we inferred work experience, qualification and non-monetary incentives as strong determinants of current level of involvement of RKS members in governance and management of health units.

**Conclusion:**

Poor knowledge/expectation of RKS members was diluting the decision making process at DMHUs. There is an urgent need to improve their knowledge, understanding and expertise in areas of governance and management practices. A locally-monitored and time-bound capacity building plan could achieve this. Yearly resource allocation for drug procurement needs revision. Specific eligibility criteria based on work experience and qualification may be fixed for RKS membership. Further research may focus on identifying the underlying individual and systemic factors behind such large PD-NPD differences.

## Background

Decentralization is the process of transferring power, authority, resources, functions and service delivery responsibilities from the central government to the lower-level institutions in a political administrative structure [1–5]. ‘Power’ is the ability to influence people, while ‘authority’, power conferred for a purpose. In the context of governance of public health systems, the latter is more often referred to than the former. Decentralization or local decision-making, as is often understood, is recognized as an important means of improving service delivery [6–8]. Improved efficiency and effectiveness, accountability, responsiveness, community participation, service integration and intersectoral coordination are considered as the key benefits of decentralization. Further, it is argued that shared governance would be knowledge-based; foster decision making at the point of service, improve direct communication between the clients and decision makers, and ensure accountability [9]. However, the evidences generated on the impact of such a reform are inconclusive [10–19].

The ‘health system strengthening’ approach has gained momentum in the last decade. Many national governments and even global health institutions have started investing in systems strengthening [20–25]. Evidences indicate that the health system’s performance in achieving the objectives of efficiency, quality and equity is contingent upon the width of ‘decision space’ at the local level. The functional areas of finance, service autonomy, recruitment rules, access rules and departmental rules normally have very narrow ‘decision space’ at local level. On the other hand, the administrative environment comprises of factors, such as, support for supervisors, enabling work environment and efficient funds flow. Decentralization is considered an effective governance mechanism to promote health system performance [26–29]. The thin line of difference between governance and management is explained in terms of the roles, focus and outputs. The former is related to visioning and policy development, while the latter is mainly day-to-day implementation.

In the Indian context, the high burden of malnourishment among children, high mortalities and other pregnancy-related complications brought back the focus to improving access to care through establishment of a wide network of public health facilities, and implementation of several outreach programs. Such efforts got a boosting after the formal launching of National Health Mission (NHM), earlier termed NRHM, in April 2005 [20, 30, 31]. Universal access to affordable, equitable, and quality health care became the key objectives of public health system. Improved funds availability, ready-to-use infrastructure, higher institutional standards, availability of trained human resources, and decentralized governance became the immediate goals. In this context the historical 73rd and 74th Constitutional Amendments (1992) conferred more powers to the Gram Panchayats (GP) for rural- and Municipalities/Notified Area Councils (NAC) for urban/peri-urban areas, respectively. Many decision-taking responsibilities were devolved to people’s representatives [32]. In Odisha, the formation in 1998 of the state health & family welfare society and the amalgamation of district health & family welfare societies in 1999 were key milestones. Subsequently, for public service delivery health institutions, such as, hospitals and health care centres, Rogi Kalyan Samiti’s (RKS) were formed as institutions of local decision making to take the public health system agenda forward.

Each public health institution in Odisha from medical college to primary health centres (PHC) has a RKS as to facilitate community control, ensure quality enhancement, comply with minimal quality benchmarks, and foster local accountability and transparency in governance [24, 25, 33]. The RKS comprises of health service providers, officials in administrative/managerial role, elected representatives of panchayat raj institutions (PRI), and officials from other departments (including independent members), though such categories are neither tightly compartmentalized, nor mutually exclusive. Broadly, the functions of RKS can be classified into five thematic domains: a) Governance (accountability, responsiveness and transparency); b) Infrastructure (construction, and maintenance, purchase and out-sourcing); c) Human resources management (hiring, transfer and training of staff); d) Financial resource management (cost-cutting measures, resource generation); and e) Quality improvement (supervision, modernization, quality assurance and accreditation) [33].

Earlier studies have identified numerous demand-side factors as important barriers to healthcare utilization, but there is scant literature on the current state of affairs with regard to the functioning of local governance institutions in public health sector [34] Authors have further argued that good governance and management of health services at peripheral level are strongly associated with improved population health outcomes [35–37]. Thus it is important to examine the perception of key stakeholders about the nature and process of local self-governance. This study focused on examining the roles, involvement and experiences of RKS members in the functioning of peripheral health units in Odisha. We assessed the knowledge, perception and practices of RKS members about their own ability and willingness to address local health systems related issues, such as, recruiting staff, generating and using funds, strengthening drug procurement and logistics supply, developing infrastructure, and organizing training for the staff. The study would not only identify the gaps but also would substantially inform the policy makers, because ultimately it is the government that decides on the policies, controls and distributes resources, and brings about reforms.

### Objectives and research questions

The study aimed to assess the knowledge, perception and experiences of local decision makers about their own abilities, training status, roles, and involvement in the local governance of health units for effective functioning and service delivery. It focused on exploring their specific roles on governance, infrastructure development, human resources management, financial management and quality of service delivery. Further, we aimed to examine the perceptual and functional differences between priority and non-priority district set-ups; and to identify predictors for involvement of RKS members in local governance of health units.

## Methods

### Working definitions

Devolution refers to the legal transfer of power to democratically elected local political organs, independent of the central government. Deconcentration is handing over some of its authority to the administrative local offices of the ministry, responsible for health. Delegation refers to the transfer of defined managerial and administrative functions and responsibilities to institutions that are outside of the central government. These institutions can be indirectly controlled by the health ministry [13, 38–42]. Individual factors in this study included work experience, qualification, interest for future involvement, current training status and interest for future training; under organizational factors we included district category, relationship with other RKS members, frequency of conducting RKS review, power/authority, community demands, monetary incentives, good leadership, non-monetary incentives, and other work-related factors.

### Study setting

The peripheral public healthcare delivery system operates at four hierarchical levels: sub-centres (SC), primary health centers (PHC), community health centers (CHC), and sub-divisional hospitals (SDH) and district headquarters hospitals (DHH). CHCs serve a population of 80,000-120,000; PHCs cater to about 20,000-30,000 population, while SCs, approximately 5,000. SCs are manned with one auxiliary nurse and midwife (ANM) and, sometimes, another additional ANM to provide essential primary care. PHCs are stipulated to have six inpatient beds, one medical officer and 16 paramedical and other staff. The activities involve curative, preventive, and promotional health care. PHCs are expected to be equipped to provide 24/7 normal and assisted deliveries, ante-natal care, postnatal care, newborn care, family planning, and full child immunizations. CHCs have multiple doctors and/or specialists, about 20–30 inpatient beds and a committed information manager [43]. The DHH is the highest service delivery point at district level, with about 100–500 beds, multiple specialists and services. The state has 6688 SCs, 1279 PHCs, 120 other hospitals, 231 CHCs, 22 SDHs, 32 DHHs and 3 Medical Colleges (MC) [44]. Each institution from PCH to MC has a registered RKS. With an average of 12 RKS members per institution, the state is estimated to have about 20200 RKS members.

### Sampling

We adopted a multi-stage stratified random sampling technique. On the basis of pre-existing government of Odisha zoning of the state, we geographically clustered 30 districts in to three zones – Central, Northern and Southern. Each zone covered ten districts. Second, the infant mortality rate (IMR) was taken as a proxy indicator of health system performance; use of process indicators was avoided in view of poor validity. As per the IMR figures of annual health survey (AHS) report of 2011, from each zone, the best ranked and the worst ranked district was selected as the primary sampling unit (PSU). Three districts from three zones having highest IMR constituted the PD and three having lowest IMR were categorized as NPD. Service delivery institutions in the sample districts constituted the secondary sampling units (SSU). To avoid the urban-rural bias, from each sample district, the DHH was invariably included in the study sample. Two CHCs, one each from urban and rural areas; and two PHCs, one each, under the administrative jurisdiction of the sample CHCs were selected randomly. Thus, 30 institutions/health units (1 DHH, 2 CHCs and 2 PHCs per district) spread across six sample districts constituted the sites for data collection. Open Epi software (http://www.openepi.com/SampleSize/SSPropor.htm) was used for sample size calculation for respondents of two groups of districts. The sampling universe constituted all RKS members. We used the following formula for sample size calculation: *n* = [DEFF*Np (1-p)]/ [(d^2^/Z^2^_1-α/2_*(N-1) + p*(1-p)]. With hypothesized 70 % +/− 10 % frequency of outcome factor (current involvement in management of the health unit) in the population (*p*), Confidence limits of 10 % of 100 (absolute +/− %) (*d*), and design effect of 1, we estimated that 57 samples from each category of districts would be sufficient. In total 112 respondents were interviewed.

### Data collection

A semi-structured interview schedule was developed in English, field-tested and translated into local language. It contained 65 close-ended questions and 8 open-ended questions. The schedule contained questions on five broad domains: nature of decision making with regard to local governance, human resources management, funds management, infrastructure development, and quality control; we also elicited responses on regularity of holding RKS meetings, involvement of RKS members in planning, monitoring and supervision of programs, their current training status and training needs. Permission was obtained from the health & family welfare department, government of Odisha for conducting this study. Subsequently the study was approved by an independent Ethics Committee of IIPHB. Two field investigators were hired, trained and engaged in data collection. The researcher visited about 20 % sites for conducting interviews and monitoring quality of data collection. We obtained written consent from each respondent prior to the interview. Data collection was done during Sep 2013-June 2014.

### Data analysis

The data were entered into MS Access 2007 (Microsoft Inc., Redmond, WA, USA), after cleaning and validation. We used SPSS 20.0 (SPSS Inc) for data analysis. Descriptive and inferential statistics were used during data analysis. *P* value of ≤ 0.05 was considered significant and of ≤ 0.001 as highly significant. Mean, median and standard deviation (SD) were used to analyze respondent profile, frequencies and cross tabulations. All continuous variables were described in-terms of Mean (+/− SD), Median and their Range. Categorical variables were presented in frequency tables. Associations between the two categories of districts (priority and non-priority) were analyzed for each dependable variable. Chi square test of significance was used for nominal and ordinal data. We felt it appropriate to conduct an independent *t*-test for interval data as well as all ranked responses as those were converted into scores for quantitative comparison. A composite of individual factors with a score range of 0–16 was developed for 16 designated activities in four work-related domains: a) current level of involvement in management of the local health unit; b) interest for future involvement; c) training status against each activity; and d) interest for future training. We assessed the perception of respondents in a Likert’s scale of 1–5 (1 = least and 5 = most) about the importance of organizational factors, such as, power, money, leadership, community demands, non-monetary incentives and other work-related factors in improving performance of the health units. In the last section of analysis, we used a linear regression model to identify predictors of involvement of RKS members in local governance. In the model, we included current involvement in management of the local health unit as dependent variable (DV); and the following as independent variables (IV): individual factors (work experience, qualification, interest for future involvement, current training status and interest for future training); and organizational factors (district category, relationship with other RKS members, frequency of conducting RKS review, power/authority, community demands, monetary incentives, good leadership, non-monetary incentives, and other work-related factors).

## Results

Fifty five respondents from PD and 57 from NPD participated in the study. The socio-demographic profile of respondents in both groups of districts was found to be comparable. The mean age of respondents in PD and NPD districts was (41.1 ± 11.0) and (40.7 ± 11.5) years, respectively (*p* = 0.841). Male respondents in PD constituted 72.7 % as against 71.9 % in NPD (*p* = 0.925). Majority of the respondents were having experience of 2–5 years (68.7 %); about one-fifth respondents (14.3 %) had less than 1 year of experience, whereas those having > 6 years of experience constituted 17 % (*p* = 0.08). About three-fourth of respondents had at least graduation level education, while 2.7 % were educated up to 10^th^ standard. Health service providers constituted the maximum proportion of respondents (44.6 %), followed by officials in administrative/managerial role (37.5 %); elected representatives and officials from other departments (including independent members) constituted about one-tenth, each (8.9 %); no significant differences were observed in the composition of respondents between both groups of districts. When asked to rank their relationship with other RKS members in a three point scale, majority (61.5 %) of respondents ranked it as ‘excellent’ (PD = 73.6 % vs. NPD = 50 %; *p* = 0.041) and ‘very good’ (35.8 %).

### Governance

As compared to PD, higher proportion of respondents from NPD was involved in district plan preparation (NPD = 78.9 % vs. PD = 58.5 %) and trained in preparing the plan (NPD = 47.4 % vs. PD = 27.5 %). On the other hand, the findings were converse for variables, such as, participation of representatives from other departments (PD = 96.9 % vs. NPD = 45.5 %), inclusion of activities of other sectors (PD = 70.8 % vs. NPD = 41.8 %) and regular review of the progress (PD = 85.7 % vs. NPD = 90.2 %). All the above mentioned differences were statistically significant. Nine out of 49 respondents in PD and 6 out of 56 respondents in NPD responded positively when asked as to whether or not they could establish local priorities different from the centrally sponsored schemes while preparing the annual plan (Table 1). On further probing about what they had included as ‘different’ than ‘local priorities’, only 4 from PD and 2 from NPD responded. Fluorosis control, malaria and dengue control, provision to address water shortage and management of malnutrition were cited as the special programs.

**Table 1:** Opinion, perception and practices related to governance

Attributes	District classification						P
	Priority		Non-priority		Total		
	No.	%	No.	%	No.	%	
Are you involved in development of district / sub-district plan of the current year?							
Yes	31	58.5	45	78.9	76	69.1	*p* = 0.020*
No	22	41.5	12	21.1	34	30.9	
Total	53	100	57	100	110	100	
Are you trained in preparation of district/block operational plans (PIP) development?							
Yes	14	27.5	27	47.4	41	38	*p* = 0.033*
No	37	72.5	30	52.6	67	62	
Total	51	100	57	100	108	100	
Could you establish local priorities different from the centrally sponsored schemes?							
Yes	9	18.4	6	10.7	15	14.3	*p* = 0.264
No	40	81.6	50	89.3	90	85.7	
Total	49	100	56	100	105	100	
Do officials from other departments participate in formulating district/block health plan?							
Yes	40	76.9	25	45.5	65	60.7	*p* < 0.001**
No	12	23.1	30	54.5	42	39.3	
Total	52	100	55	100	107	100	
Do you include activities of other sectors in district/block health plan?							
Yes	34	70.8	23	41.8	57	55.3	*p* = 0.003*
No	14	29.2	32	58.2	46	44.7	
Total	48	100	55	100	103	100	
How frequently are review/monitoring meetings for health activities conducted?							
Weekly	3	6	4	7.1	7	6.6	*p* = 0.069
Monthly	14	28	26	46.4	40	37.7	
Quarterly	32	64	22	39.3	54	50.9	
End of year	0	0	0	0	0	0	
Irregularly	1	2	4	7.1	5	4.7	
Total	50	100	56	99.9	106	99.9	

* Significant. ** Highly significant

### Human resource management

We studied the knowledge, perception and experience of RKS members on human resources management (HRM) practices. We included questions related to postings, transfers and suspensions; frequency and approval of such decisions; role of state officials in such decisions; the amount of autonomy at local level; and hiring practices. Perception about the working environment and training needs were assessed separately. About 42 % decision makers in PD as against 33 % in NPD had proposed transfer or suspension of service providers in the last 2 years (Table 2). There were significant inter-category differences with regard to ‘trainings requested’ and ‘trainings conducted’ parameters - higher proportion of respondents from NPD had requested for and received training, while the need for capacity building was higher in PD.

**Table 2:** Opinion, perception and practices related to human resource management

Item	District classification						p
	Priority		Non-priority		Total		
	No.	%	No.	%	No.	%	
Did the RKS request for creation of new posts in the last year?							
Yes	12	23.5	13	25.5	25	24.5	*p* = 0.818
No	39	76.5	38	74.5	77	75.5	
Total	51	100	51	100	102	100	
If yes, were all the requests approved?							
Yes, all were approved	3	30	1	7.7	4	17.4	*p* = 0.179
Some were approved	2	20	7	53.8	9	39.1	
None was approved	5	50	5	38.5	10	43.5	
Total	10	100	13	100	23	100	
Nature of support received from seniors in the district/block?							
Very cooperative	29	74.4	48	90.6	77	83.7	*p* = 0.024*
Supportive of health program	4	10.3	2	3.8	6	6.5	
Not concerned at all	1	2.6	3	5.7	4	4.3	
Others	5	12.8	0	0	5	5.4	
Total	39	100	53	100	92	100	
Nature of relationship with subordinates?							
Very good	26	54.2	28	50	54	51.9	*p* = 0.689
good	18	37.5	25	44.6	43	41.3	
Bad	4	8.3	3	5.4	7	6.7	
Very bad	0	0	0	0	0	0	
Total	48	100	56	100	104	100	
Do the staff in your opinion require any kind of training?							
Yes	43	82.7	50	89.3	93	86.1	*p* = 0.322
No	9	17.3	6	10.7	15	13.9	
Total	52	100	56	100	108	100	
Was any special training program requested in the last financial year plan?							
Yes	8	17.8	36	66.7	44	44.4	*p* < 0.001**
No	37	82.2	18	33.3	55	55.6	
Total	45	100	54	100	99	100	
Was any special training conducted in the last financial year?							
Yes	6	16.7	30	76.9	36	48	*p* < 0.001**
No	30	83.3	9	23.1	39	52	
Total	36	100	39	100	75	100	

* Significant. ** Highly significant

### Financial management

Variables such as, funds allocation, resource generation through user fees, use of additional funds, and vacancy in accounts section were assessed (Table 3). About half of the respondents (50.9 %) indicated that the amount of funds allocated under NHM was ‘manageable’, one-fourth (25.9 %) felt it was ‘grossly insufficient’; whereas 18.5 % respondents felt it was ‘sufficient’ or ‘more than adequate’. About 92 % respondents said user fees was charged for various services in their respective health units. When further probed as to how such collected fees were used, responses included the following: minor repair of infrastructure, fuel for generator, X-ray maintenance, purchase of Ultrasonography/Ecocardiogram rolls, reagents for pathology test and emergency drugs. Further, 40.7 % respondents said that the district proposals got approvals in their original forms, whereas about 21 % respondents replied that they never received any feedback from higher up. Significantly higher proportion of respondents from NPD said they received the approved proposal in its original form, as compared to PD. About frequency of audits conducted in the institution, 92 % respondents in PD and 63 % in NPD said audits were conducted quarterly – this difference was also statistically significant. Fifty percent of respondents said they requested for additional funds, last year, and on further probing, 38.8 % respondents informed that they requested additional funds for purchase of drugs.

**Table 3:** Opinion, perception and practices related to financial management

Items	District classification						p
	Priority		Non-priority		Total		
	No.	%	No.	%	No.	%	
The amount of funds allocated under NHM to the RKS?							
More than enough	0	0	2	3.6	2	1.9	*p* = 0.100
Sufficient	12	23.1	7	12.5	19	17.6	
Manageable	25	48.1	30	53.6	55	50.9	
Insufficient	15	28.8	13	23.2	28	25.9	
Grossly insufficient	0	0	4	7.1	4	3.7	
Total	52	100	56	100	108	100	
Is user-fees being imposed in the institution (health unit)?							
Yes	45	88.2	53	94.6	98	91.6	*p* = 0.233
No	6	11.8	3	5.4	9	8.4	
Total	51	100	56	100	107	100	
What happens to your request for additional requirements/needs, at state level?							
No feedback received	11	27.5	8	15.7	19	20.9	*p* < 0.001**
Got approved as it was	6	15	31	60.8	37	40.7	
Got approved with reduction of budget	7	17.5	4	7.8	11	12.1	
Never gets approved	5	12.5	2	3.9	7	7.7	
Others	11	27.5	6	11.8	17	18.7	
Total	40	100	51	100	91	100	
What is the frequency of financial audit?							
Quarterly	46	92	34	63	80	76.9	*p* < 0.001**
Half yearly	1	2	5	9.3	6	5.8	
Yearly	2	4	0	0	2	1.9	
Don’t know	0	0	1	1.9	1	1	
Others	1	2	14	25.9	15	14.4	
Total	50	100	54	100	104	100	
Any additional funds requested last year?							
Yes	26	54.2	25	46.3	51	50	*p* = 0.427
No	22	45.8	29	53.7	51	50	
Total	48	100	54	100	102	100	
If ‘yes’, for what purpose?							
Drugs	8	34.8	11	44	19	39.6	*p* = 0.036*
Equipment	5	21.7	2	8	7	14.6	
Personnel	6	26.1	1	4	7	14.6	
Other purposes	4	17.4	11	44	15	31.3	
Total	23	100	25	100	49	100	

* Significant. ** Highly significant

### Functioning of health units

Less than a tenth of respondents (9.7 %) replied affirmatively about initiation of new programs for innovative health services. Health system strengthening, free medicines, health camps and awareness programs were cited as new initiatives (Table 4). Further, with regard to the question of new strategies for service delivery after formation of RKS, 82.8 % respondents replied. To an open-ended question as to list the new programs/services that had been introduced by the RKS in last 1 year, we got various subjective and irrelevant responses. With respect to the role of state officials in solving local problems, more than half of respondents (58.3 %) felt that state directives were useful for solving local problems – this proportion was significantly higher in NPD as compared to PD (NPD = 78.4 %; PD = 35.6 %; *p* < 0.001). With regard to their roles, satisfaction level and perception about importance of work-related factors, about 87 % respondents were satisfied with their role in the local governance of the health units. However, this was significantly higher (94.3 %) in PD as compared to NPD (80.7 %). Further, when asked as to how satisfied they were about their contribution to the improvement of functioning of local institutions, almost all respondents (98.2 %) were ‘satisfied’. Almost all respondents (98 %) in PD and more than three-fourth (80.7 %) in NPD opined that local decision making helped in improving the performance of local institutions – this difference between PD and NPD was significant.

**Table 4:** Opinion, perception and practices related to health unit functioning

Items	District classification						p
	Priority		Non-priority		Total		
	No.	%	No.	%	No.	%	
Did RKS initiate any new programs in the institution in last three years?							
Yes	5	10.6	5	8.9	10	9.7	*p* = 0.770
No	42	89.4	51	91.1	93	90.3	
Total	47	100	56	100	103	100	
Did RKS develop innovative methods for providing health services in last three years?							
Yes	35	83.3	42	82.4	77	82.8	*p* = 0.901
No	7	16.7	9	17.6	16	17.2	
Total	42	100	51	100	93	100	
Are the state directives helping to solve the local problems?							
Yes	16	35.6	40	78.4	56	58.3	*p* < 0.001**
No	29	64.4	11	21.6	40	41.7	
Total	45	100	51	100	96	100	
Are you happy with the role in management of the local institution?							
Yes	50	94.3	46	80.7	96	87.3	*p* = 0.032*
No	3	5.7	11	19.3	14	12.7	
Total	53	100	57	100	110	100	
Are you satisfied with contribution to the improvement of the local institutions?							
Yes	52	98.1	56	98.2	108	98.2	*p* = 0.959
No	1	1.9	1	1.8	2	1.8	
Total	53	100	57	100	110	100	
Does local decision making help in improving performance of the institutions?							
Yes	50	98	46	80.7	96	88.9	*p* = 0.004*
No	1	2	11	19.3	12	11.1	
Total	51	100	57	100	108	100	

* Significant. ** Highly significant

### Involvement and training

With respect to the frequency of their meeting with or contacting the zilla parishad (ZP) or block parishad (BP) president, it was found that about one-sixth of the respondents (15.9 %) never contacted or met the latter. Higher proportion of members from NPD (26.3 %) had ‘never met’ the president as compared to PD (4 %) and this difference was significant (*p* = 0.018). Less than one third of the respondents (31.2 %) said that they discussed health, water and sanitation issues in RKS meetings, whereas majority of respondents (68.8 %) replied that they discussed issues other than health. Further, 27.6 % respondents said they ‘never’ attended the ZP/BP meetings, as against 30.6 % who said they ‘always’ attended, while 41.8 % participants said they attended such meetings ‘sometimes’. On the issue of new health activities initiated by the ZP and/or BP in last two years, only 5.9 % respondents replied positively and 16.8 % were under ‘can’t say’ category. When asked as to ‘whether or not the local elected representatives provided funding support to the RKS, 20 % said ‘yes’ and about one-sixth were ‘not aware’ of such support – the proportion was significantly higher in NPD. In the last section, we assessed their current level of involvement and training status as to undertake the 12 designated responsibilities; it was found no significant difference with respect to the former (involvement) between PD and NPD and staggering differences with respect to the latter (training status) – those from NPD had received maximum numbers of training courses, whereas the need for training was the highest in PD.

### Factors and predictors of local governance

The mean composite scores for individual factors about involvement and training of RKS members were obtained. We found the mean score ranged from 15.12 (current involvement) to 15.88 (interest for future involvement and training); for training status, the scores was abysmally low in PD (0.76) and low for NPD (3.64) in a scale of 0–16. Further, with regard to the perception about importance of organizational factors, the mean scores for most of the factors were higher than 4 in a scale of 0–5. We conducted an independent *t*-test to examine the PD-NPD differences of perceptual means with respect to individual and organizational factors as reflected in Table 5. We found significant to highly significant difference in perception of respondents between PD and NPD on relationship with other RKS members (0.015), training status (0.003), monetary incentives (0.007), good leadership (0.003), community demands (0.010) and non-monetary incentives (0.001), and other work related factors (<0.001). Through regression analysis, we inferred factors, such as, work experience, qualification and non-monetary incentives are strongly associated with current level of involvement of RKS members in management of the health units (Table 6).

**Table 5:** Independent *t*-test for equality of means of individual and organizational factors

Independent *t*-test for equality of means							95 % CI of the difference	
Factors	District type	N	Mean	Std. deviation	Std. error mean	Sig. (2-tailed)	Lower	Upper
Age	Priority	55	41.13	11.039	1.488	.829	−3.761	4.682
	Non-priority	57	40.67	11.505	1.524			
Work experience (years)	Priority	55	3.76	1.962	.265	.434	-.442	1.022
	Non-priority	57	3.47	1.947	.258			
Relationship with other RKS members	Priority	53	4.7170	.4952	.0680	.015^a^	.0492	.4561
	Non-priority	56	4.4643	.5709	.0763			
Current Involvement	Priority	49	15.489	2.0219	.28886	.417	-.5304	1.2700
	Non-priority	50	15.1200	2.4713	.3495			
Interest for future involvement	Priority	49	15.306	3.1963	.4566	.206	−1.4801	.32316
	Non-priority	52	15.884	.70444	.0976			
Training status	Priority	46	.7609	3.3010	.4867	.003^a^	−4.7485	−4.74857
	Non-priority	51	3.6471	5.534	.7750			
Interest for future training	Priority	45	15.888	.43809	.06531	.655	-.2135	.33743
	Non-priority	52	15.826	.8794	.12195			
Power/authority	Priority	49	4.49	.845	.121	.009	.132	.919
	Non-priority	56	3.96	1.144	.153			
Monetary incentives	Priority	49	4.47	.868	.124	.007^a^	.156	.961
	Non-priority	56	3.91	1.164	.156			
Good leadership	Priority	47	4.60	.771	.112	.003^a^	.211	.981
	Non-priority	56	4.00	1.128	.151			
Community demands	Priority	47	4.51	.882	.129	.010^a^	.129	.928
	Non-priority	56	3.98	1.120	.150			
Non-monetary incentives	Priority	45	4.56	.755	.113	.001^a^	.287	1.074
	Non-priority	56	3.88	1.145	.153			
Other work related factors	Priority	39	4.77	.485	.078	<.001^b^	.479	1.242
	Non-priority	55	3.91	1.127	.152			

^a^ Significant. ^b^ Highly significant

**Table 6:** Linear logistic regression for predictors of local governance

Variables	Beta coefficient	*P* value	95.0 % CI	
			Lower bound	Upper bound
Work experience (years)	-.096	0.472	−0.363	0.17
Qualification	1.595	.001**	0.69	2.5
Interest for future Involvement	−1.137	.020*	−2.09	−0.184
Current training status	-.101	0.161	−0.243	0.041
Interest for future training	.213	0.602	−0.601	1.027
District category	.664	0.31	−0.633	1.96
Relationship with other RKS members	.648	0.183	−0.314	1.61
Frequency of conducting RKS review meetings	-.605	0.139	−1.411	0.201
Power/authority	.364	0.716	−1.626	2.353
Monetary incentives	-.972	0.315	−2.89	0.947
Good leadership	−1.057	0.356	−3.327	1.214
Community demands	-.380	0.641	−2.003	1.243
Non-monetary incentives	3.750	.018*	0.677	6.823
Other work-related factors	−1.414	0.283	−4.024	1.197

* Significant. ** Highly significant

## Discussion and conclusion

Leadership and governance is one of the six pillars of WHO-proposed building block framework on health system strengthening [21, 45]. The goal is to support a health system that aims to protect lives; prevent, treat and control diseases; and maintain population health [45]. Even though Maun et al. have questioned the success of shifting power from officials to citizens in improving the quality and efficiency of care, the outcomes of such reforms might vary in different contexts [46]. In governance of health units, the roles and responsibilities of RKS members in annual health plan preparation is a critical step towards improving effectiveness of their functioning. Whereas, concerns related to involvement of other department officials and establishing local priorities could pose serious challenges to the very existence RKS in attainment of common objectives. The authority for transfers, promotions and postings of health workforce are vested with the state government, except for periodic arrangements at district level to deal with district cadres, such as, the nursing professionals and paramedics. One possible mechanism to delegate more powers to the RKS could be through legislative route. The Society Registration Act of 1860 and provisions therein have flexibilities to perform, but power/authority and transparency in decision-making are not priorities of the Act. Responsibilities must commensurate with authority and expertise. Needless to say, one needs to adopt a cautious approach to ascertain the inherent expertise of RKS in taking rational decisions with respect to local hiring and human resource management practices - this needs further scrutiny [9].

The quality of MNH care is dependent on availability of personnel, funds and logistics support. The study findings could be used to strengthen the national-level policy for improving the quality of MNH care at the facilities [47]. For better availability and management of funds, the guidelines have been circulated to all States/Union Territories (UT) by government of India. Funds to the tune of INR 100,000 per PHC, 200,000 per CHC/SDH and INR 500,000 per DHH are released to the concerned RKS, every year. On the one hand the RKS members are seeking more funding support from the state, but the difficulties in utilization of such funds are often discussed in various platforms, including review meetings. Moreover, irregular audits and irrational request for additional funds raise serious questions about the ability of RKS in planning, implementing and monitoring development activities in compliance with the overall financial guidelines. RKS is considered as a local self-governing institution to improve the local management responses and in turn, strengthening health system preparedness for improved service delivery. However, we find that decentralized decision-making by RKS does not have a commensurate collective knowledge, experience and expertise for governing health units. Individual factors, such as, experience, qualification and non-monetary incentives could play critical role in ensuring involvement of RKS members in local self-governance.

The District Level Household Survey-3 (DLHS-3) report has pointed out that the constitution and utilization of ‘untied RKS funds’ in the CHC and DHH had been relatively successful; however, the implementation of programs by RKS proved problematic at the PHC level. We find higher sense of satisfaction about the involvement of members in decision making, but porr training status and higher need for training. Thus, poor knowledge and understanding of possible newer service delivery strategies, poor information about their responsibilities, and non-responsiveness to the patients’ rights could possibly act as underlying factors for poor functioning of RKS at PHC level. In fact, some of the health units didn’t have the citizen’s charters displayed, as observed by the researcher during data collection. On the other hand, the members had high level of self-satisfaction about their contribution to the health system. These findings are reflective of low level of expectation, poor role clarity, and may be, of a sense of complacency. A recent study has indicated that inadequate support systems for capacity building and training of local decision makers are constraints which weakened the impact of RKS [48]. Our study confirms the earlier similar inferences. Periodic trainings need to be considered as a potential solution, and provision of hand-holding support to the RKS may be envisaged as a long-term option to achieve the overall objectives of strengthening governance of health units.

Majority of the respondents acknowledged the importance of ability to plan and spend the budget with higher flexibility; ability to initiate innovative health service programs, to hire contractual staff, and to be able to set district priorities for ensuring effective local choice. Irregular meeting schedules and erratic decision-making processes, on the other hand, could act as serious systemic barriers to effective shared governance in the DMHUs [49, 50]. About the perception on importance of organizational factors, the mean scores for most of the factors were higher than 4 in a scale of 0–5 – this is in conformity with existing evidences about Herzberg’s motivators and hygiene factors [51]. The gap between training needs and trainings offered to the RKS members is very wide which could be cemented through a locally-monitored capacity building plan – this could not only help in improving the knowledge and understanding of the RKS members, but also create an enabling environment for improving utilization of services. Proper grievance redressal system (e.g., mandatory display of citizens’ charter, complaint box) may be ensured in order to improve involvement of patients and the community. The functioning of RKS at the PHC and CHC level needs special emphasis because often these health units act as the first point of contact between service seekers and service providers.

Setting the agenda of meetings in advance, following consultative process during meetings, and provision of hand-holding support by the higher level institutions may be considered as potential strategies to overcome these problems. Conducting regular and productive review meetings by the RKS was considered a major challenge because of poor role clarity, non-availability of members and their conflicting priorities. The state government may develop a mechanism to frame stronger eligibility criteria, including work experience and qualification to enter RKS governing body, and provide non-monetary incentives to the members in order to strengthen their involvement in governance of health units.

Using a large cross-sectional institution-level dataset, this study contributes to better realization of some of the supply-side factors – it assessed the situation in terms of knowledge, perception, practices and functioning of RKS as an institution of local decision making in public health sector. It also identified individual and organizational factors contributing to RKS functioning, and examined the factors. The study also raises concerns on prioritization of resource allocation to meet the training needs of stakeholders. However, whether or not more empowerment of local RKS bodies would result in improved utilization of health services, and how well to address some of the inter-personal barriers to effective functioning of these institutions, would need further research. As other studies have pointed out, an important aspect of further investigation is the issue of formal and informal coordination mechanisms being followed at various levels that control the rules of the game [21]. A pilot implementation research may provide answers. Further, the effects of decentralized governance may be studied from the perspective of patients, service providers and the community at large.

## Limitations

This study has several strengths. To our knowledge, this is the first study on assessing the knowledge, experience and opinion of local decision makers in health sector in India. The findings could contribute to the scant literature on the subject. An empowered RKS could only attain the objectives of high quality service delivery in an accountable and transparent manner. Data collection from six 6 districts makes the study geographically representative of the state. However, with the cross-sectional nature of study design, pinning down the direction of association was difficult. A mixed methods study using reliable and valid health service routine data from these facilities could be helpful in supporting or refuting perceptions of respondents. More detailed in-depth interviews could have explored the reasons for the differences between PD and NPD health facilities.
